# Supplementary material for: Profiling the miRNA from Exosomes of Non-Pigmented Ciliary Epithelium-Derived Identifies Key Gene Targets Relevant to Primary Open-Angle Glaucoma
Source: Antioxidants (Basel). 2023 Feb 7;12(2):405. doi: 10.3390/antiox12020405 (PMC9952153; doi:10.3390/antiox12020405)
Supplement: Supplementary file 1 [file antioxidants-12-00405-s001.zip › antioxidants-2167083-supplementary.pdf]

Report for Cell Line Authentication

The Cell line authentication test was performed at the Genomics Center of Biomedical Core Facility, Technion.

The test was performed using the Promega GenePrint 24 System in order to determine short tandem repeat (STR) profile of 23 loci plus Amelogenin for gender determination (X or XY). In addition, the male-specific DYS391 locus is included to identify null Y allele results for Amelogenin. DNA sample from the kit (2800M Control DNA) was included in the analysis and served as positive control for the PCR step. No DNA template was also included as negative control.

The results were analyzed using the 3500xl Genetic Analyzer (Life Technologies) and GeneMapper IDX software. Allelic ladder was included in the run. Importantly, the 3500xl system is calibrated once a year and spectral calibrated once every 3 months by authorized person.

This report includes the STR results obtained for your cell line together with the available STR profile. It is important to note that not all STRs that are included in the kit are published. Usually, we compare the results to the Cellosaurus, a cell-line knowledge resource (Bairoch A., J. Biomol Tech, 2018).

The interpretation of results is based on allele calls for all 24 loci and should aid determine whether your cell line is indeed the cell line in question or has profile characteristics which indicate a change, contamination or misidentification.

The raw data appears in Appendix A.

Results

Cell line ID: NPCE (ODM-2)

Marker	NPCE (ODM-2)
**AMEL**	X,Y
**D3S1358**	15,17
**D1S1656**	13,14
**D2S441**	11,13.3
**D10S1248**	15,16
**D13S317**	12,14
**Penta E**	8,13
**D16S539**	12,13
**D18S51**	17,18
**D2S1338**	19,22
**CSF1PO**	10,11
**Penta D**	10,13
**TH01**	7,8
**vWA**	16,17
**D21S11**	28,30
**D7S820**	12
**D5S818**	10,12
**TPOX**	8,12
**DYS391**	10
**D8S1179**	12,15
**D12S391**	20,24
**D19S433**	12,13
**FGA**	23,25
**D22S1045**	11

Summary

There is no published profile for line NPCE (ODM-2).
A search through the database gave no results, meaning there is no known line with this profile.
In light of these results, it seems that the tested cell line is unique however, we cannot state whether the tested line is indeed NPCE (ODM-2).

Date: 12.11.19

Doron Fogel, MSc, Application specialist **Signature:** ______________

Liat Linde, PhD, Head **Signature:** ______________

We would appreciate the acknowledgment of the Genomics Center of Biomedical Core Facility, Technion in your published work that utilizes the cell line above.

Appendix A

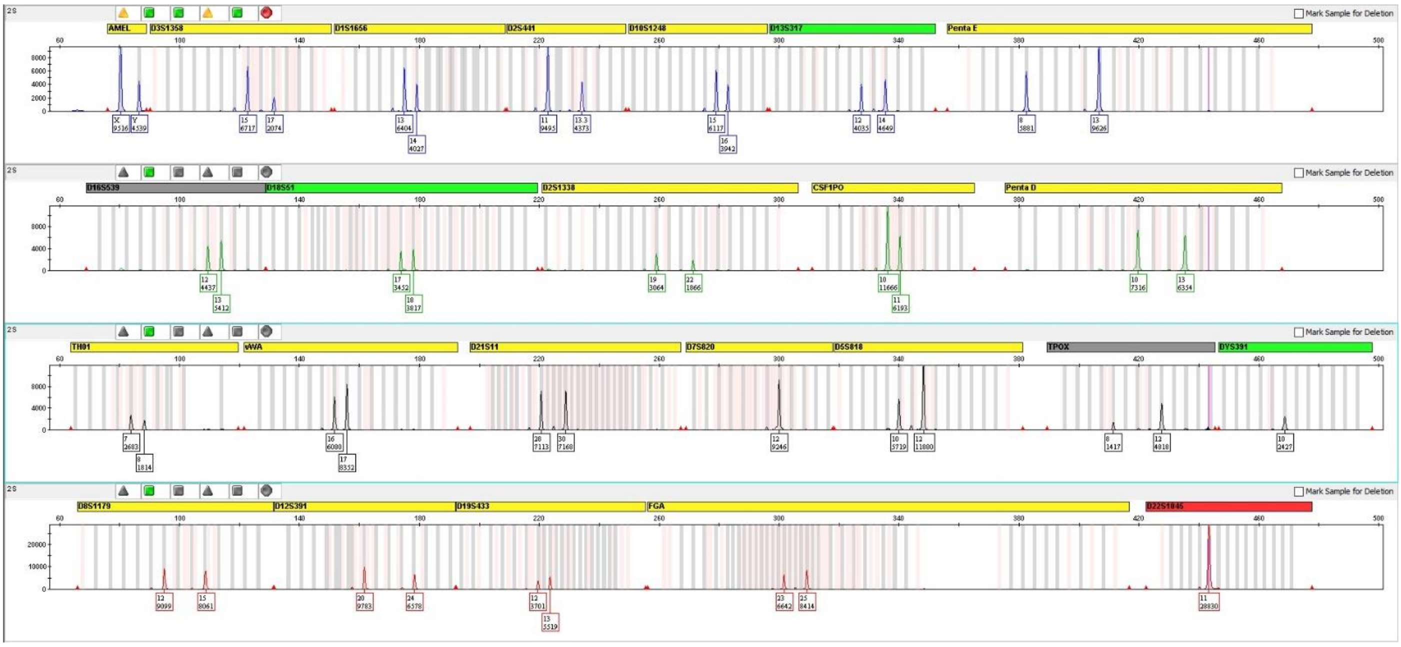
AMEL
D3S1358
D1S1656
D2S441
D10S1248
D13S317
Penta E
D16S539
D18S51
D2S1338
CSF1PO
Penta D
TH01
vWA
D21S11
D7S820
D5S818
TPOX
DYS391
D8S1179
D12S391
D19S433
FGA
D22S1045
Mark Sample for Deletion
